# Association between ambient air pollution and age-related macular degeneration: a meta-analysis

**DOI:** 10.1186/s12886-024-03465-y

**Published:** 2024-04-30

**Authors:** Jiali Wu, Yuzhu Zhang, Xian Xu

**Affiliations:** 1 Department of Ophthalmology, Shanghai General Hospital, Shanghai Jiao Tong University School of Medicine, Shanghai, China; 2 Department of Ophthalmology, Shanghai General Hospital, Shanghai Jiao Tong University School of Medicine, National Clinical Research Center for Eye Diseases, Shanghai Key Laboratory of Ocular Fundus Diseases, Shanghai Engineering Center for Visual Science and Photomedicine, Shanghai, 200080 China

**Keywords:** Ambient air pollution, Age-related macular degeneration, Meta-analysis

## Abstract

**Background:**

Several epidemiological studies have investigated the association between ambient air pollution and age-related macular degeneration (AMD). However, a consensus has not yet been reached. Our meta-analysis aimed to clarify this association.

**Methods:**

Databases, including PubMed, EMBASE, and Web of Science, were searched for relevant studies from 01 January 2000 to 30 January 2024. English-language, peer-reviewed studies using cross-sectional, prospective, or retrospective cohorts and case–control studies exploring this relationship were included. Two authors independently extracted data and assessed study quality. A random-effects model was used to calculate pooled covariate-adjusted odds ratios. Heterogeneity across studies was also tested.

**Results:**

We identified 358 relevant studies, of which eight were included in the meta-analysis. Four studies evaluated the association between particulate matter less than 2.5 μm in diameter (PM_2.5_) and AMD, and three studies explored the relationship between nitrogen dioxide (NO_2_) or ozone (O_3_) and AMD. The pooled odds ratios were 1.16 (95% confidence interval [CI]: 1.11–1.21), 1.17 (95% CI: 1.09–1.25), and 1.06 (95% CI: 1.05–1.07), respectively.

**Conclusion:**

Current evidence suggests a concomitant positive but not causal relationship between PM_2.5_, NO_2_, or O_3_ and AMD risk.

**Supplementary Information:**

The online version contains supplementary material available at 10.1186/s12886-024-03465-y.

## Background

Age-related macular degeneration (AMD) is one of the leading causes of blindness in the aging population [1–4]. Late-onset AMD can be classified into dry and wet forms. Although intravitreal injection of anti-vascular endothelial growth factor agents is the first-line therapy for AMD, not all patients benefit from this treatment [5]. The underlying mechanism is multifactorial and remains unclear [6]. Recent evidence has suggested a potential influence of ambient air pollutants on AMD risk [7, 8]. Thus, further investigation of this correlation would be clinically meaningful.

Many compounds comprise ambient air pollution, including nitrogen dioxide (NO_2_), carbon monoxide (CO), sulphur dioxide (SO_2_), ozone (O_3_), and particulate matter less than 2.5 and 10 μm in diameter (PM_2.5_ and PM_10_, respectively) [9]. Air pollution is a major contributor to the global disease burden and associated with health hazards [10–12]. Prior epidemiological studies have demonstrated that ambient air pollution is a potential risk factor for AMD [13, 14]. For example, a national cross-sectional study in China reported a significant positive association between PM_2.5_ and AMD. For PM_2.5_, compared with the lowest quartile, the odds ratios (ORs) and 95% confidence intervals (CIs) across increasing quartiles were 0.828 (0.674, 1.018), 1.105 (0.799, 1.528), and 2.602 (1.516, 4.468) [14]. However, conflicting findings were observed on the association between AMD and NO_2_ or O_3_ [7, 13]. Further investigation is needed to clarify the correlation between ambient air pollution and AMD. Therefore, we conducted this meta-analysis to report a more robust and reliable outcome.

## Methods

### Search strategy

This meta-analysis was conducted following the Meta-Analysis of Observational Studies in Epidemiology guidelines. The PubMed, EMBASE, and Web of Science databases were searched for literature using the key words ‘ambient air pollution, particulate matter, ozone, sulphur dioxide, nitrogen dioxide, or carbon monoxide’ and ‘AMD or age-related macular degeneration’ from 01 January 2000 to 30 January 2024. Furthermore, references from original studies or relevant reviews were manually searched to identify other relevant studies. All included studies were epidemiological investigations; the language was restricted to English. Two investigators independently retrieved and reviewed the full texts and abstracts of all related literature. Conflicts were resolved through a full-text review and discussed by two independent reviewers until a consensus was reached.

### Inclusion and exclusion criteria

The following inclusion criteria were applied: the studies (1) referred to the association between ambient air pollution and AMD; (2) contained calculable information, such as ORs, hazard ratios (HRs), the respective 95% CIs, and P-values; and (3) were English-only peer-reviewed studies using cross-sectional, prospective, or retrospective cohorts and case–control study designs. The exclusion criteria were as follows: (1) duplicate subjects; (2) abstracts, case reports, comments, reviews, and experimental study designs in laboratory settings; and (3) studies without necessary data.

### Data extraction

Two independent investigators (Jiali Wu and Yuzhu Zhang) extracted data from the included studies. The following summary data were included: last author, year of publication, air pollutant(s), statistical model, main results, study cohort, and diagnostic criteria for AMD. If articles reported the OR and P-value instead of the 95% CI, they were manually calculated.

### Bias assessment

Two independent authors assessed the study risk using the Newcastle–Ottawa Scale (NOS). Cohort studies that scored ≥ 7, 4–6, and < 4 were considered to have a low, intermediate, and high risk, respectively, whereas cross-sectional studies that scored ≥ 7, 6, and ≤ 5 were considered to have a low, intermediate, and high risk, respectively.

### Statistical analysis

Heterogeneity among studies was assessed using χ^2^-based Q-tests and inconsistency scores (I^2^). Heterogeneity was high, moderate, low, or none for I^2^ values ≥ 75%, 50–74%, 25–49%, and < 25%, respectively. Subsequently, random- and fixed-effect models were used based on the heterogeneity test results. The pooled ORs and 95% CIs were calculated to assess the risk of AMD due to ambient air pollution exposure. Regarding pooled outcome analyses, *P* < 0.05 was considered significant. All analyses were performed using RevMan 5.3. software (Review Manager, Nordic Cochrane Center, The Cochrane Collaboration, Copenhagen, Denmark).

## Results

### Study selection and characteristics

**Fig. 1:**
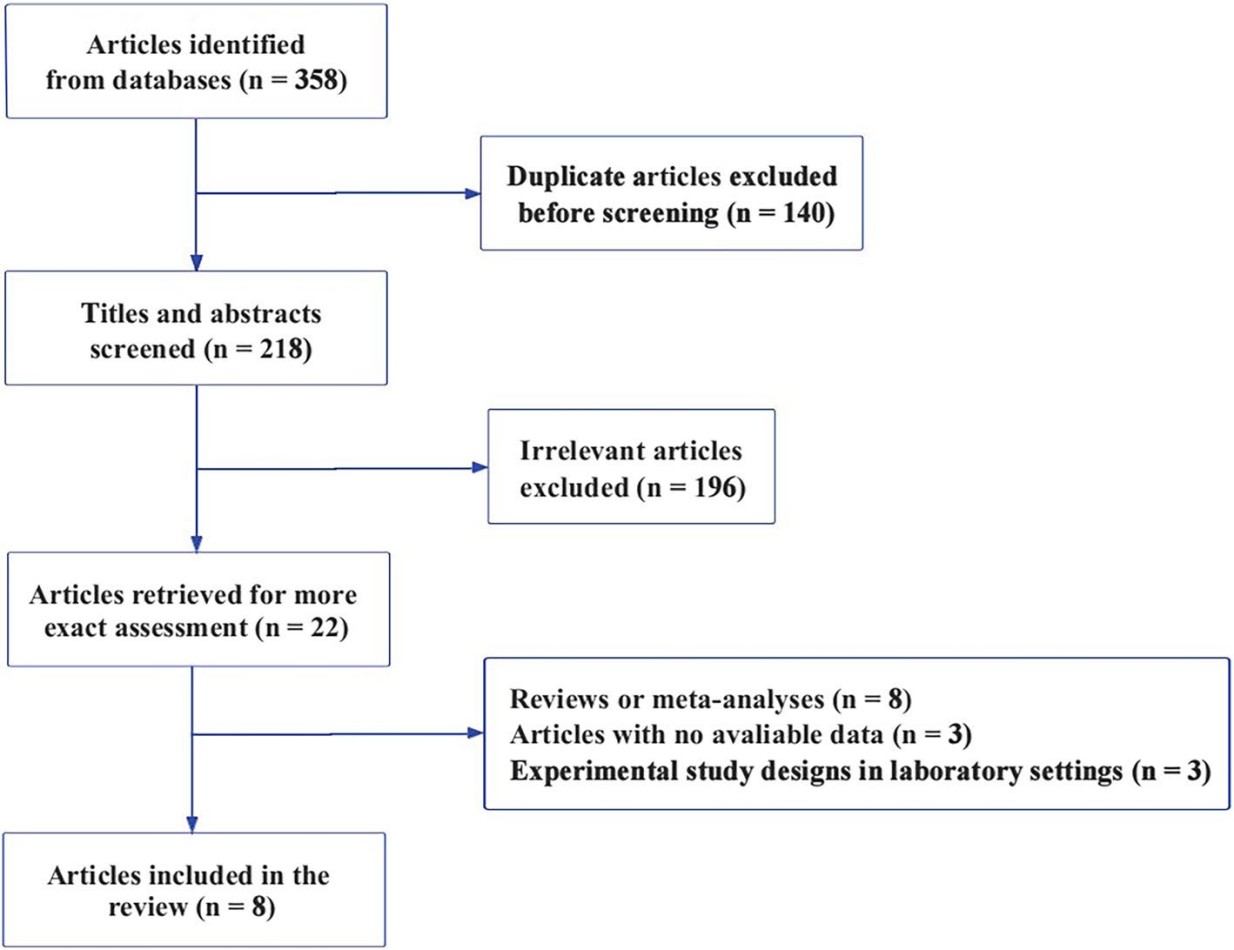
Flow diagram of the included studies

Figure 1 shows a flow diagram of the literature search. The initial search identified 358 articles from databases. After screening titles and abstracts combined with necessary full-text review, eight studies with 15,029,888 individuals were eligible for further analysis. Among these, four studies analysed PM_2.5_ [8, 13–15], and three examined NO_2_ [7, 13, 16] or O_3_ [16–18]. Two of the eight studies were longitudinal cohort studies [7, 8], while the rest were cross-sectional studies [13–18]. In the longitudinal cohort study that explored the association between the AMD risk and CO or NO_2_, 1,442 individuals among 39,819 AMD-free residents developed AMD during the study period of 11 years [7]. Moreover, 4,284,128 participants enrolled in the longitudinal cohort study evaluating the relationship between PM_2.5_ and AMD, and 12,095 AMD cases were identified during the 11-year follow-up [8].

The assessment and definition of AMD varied among studies. Among six studies applying standardised criteria for AMD diagnosis, grading was performed by at least two independent ophthalmologists to ensure the accuracy of diagnosis in three studies [8, 16, 17], whereas the other three did not describe the specific grading methodology [7, 14, 18]. Different from the standardised criteria, cases were diagnosed based on medical record review or self-reporting in two studies [13, 15]. Two AMD stages (early and late) were analysed separately in one study [16]. One study assessed AMD in patients with and without visual impairment [15]. One study explored the relationship of air pollution exposure with exudative and non-exudative AMD [18].

Table 1 summarizes the results of the individual studies in this meta-analysis. Liang et al. presented a single-pollutant model, proposing an increased risk of AMD among those exposed to the highest exposure quartile of CO (HR = 1.84; 95% CI: 1.57–2.15) and NO_2_ (HR = 1.91; 95% CI: 1.64–2.23). The risk did not increase in the second or third quartiles, indicating that moderate exposure did not lead to AMD [7]. Second, Freeman et al. reported that those exposed to higher PM_2.5_ levels were more likely to develop visually impaired AMD, as indicated by a single-pollutant regression model (OR = 1.52; 95% CI: 1.10–2.09). However, in a multipollutant model, higher exposure to PM_2.5_ merely showed a borderline association with visual impairment in AMD (OR = 1.41; 95% CI: 0.96–2.08, *P* = 0.08) [15]. In addition, single-pollutant model findings by Patel et al. demonstrated increased odds of AMD among participants exposed to higher levels of PM_2.5_ (OR = 1.08; 95% CI: 1.01–1.16). However, they did not find an association between exposure to PM_10_ (OR = 0.94; 95% CI: 0.86–1.02) or NO_2_ (OR = 0.99; 95% CI: 0.91–1.08) and AMD [13]. Furthermore, Choi et al. modelled air pollution within administrative division units, which suggested that NO_2_ (OR = 1.24; 95% CI: 1.05–1.46) and CO (OR = 1.22; 95% CI: 1.09–1.38) were risk factors for AMD, but O_3_ was associated with a decreased prevalence of AMD (OR = 0.8; 95% CI: 0.70–0.92). When air pollution was modelled as local/town units, the associations were slightly diminished. Besides, the study showed that higher levels of CO exposure led to higher prevalence of AMD [16]. However, the findings by Manookin et al., which were diametrically different from those by Choi et al., revealed that O_3_ was the only air pollution that statistically significant associated with any AMD (OR = 1.011; 95% CI: 1.003–1.019). It was demonstrated that NO_2_, SO_2_, CO and PM_2.5_ did not increase the AMD risk, whereas the exact effect size of them was not reported [18]. Both Hwang et al. and Yan et al. found a significant positive association between AMD and higher PM_2.5_ levels (HR = 1.19; 95% CI: 1.13–1.25 and OR = 2.602; 95% CI: 1.516–4.468, respectively) [8, 14]. Recently, multipollutant model findings by Sun et al. have revealed that the AMD risk was on a monotonic increasing trend with higher O_3_ concentration; the harmful effect increased rapidly after reaching a turning point of 110 μg/m^3^ (OR = 1.15; 95% CI: 1.13–1.16 and OR = 1.66; 95% CI: 1.63–1.69, respectively) increase [17]. Based on the results above, most studies revealed that higher concentrations of air pollution increased the AMD risk. However, no studies examined the correlation between the AMD risk and the exposure length.

**Table 1:** Characteristics of the studies included in the systematic review

Author, Year	Air Pollutant(s)	Statistical Model	Effect Size	Cohort	Diagnostic Criteria
Liang CL, 2019 [7]	NO_2_ and CO	Cox proportional hazard regression models	Single-pollutant models: Adjusted HR: 1.91 (95% CI: 1.64–2.23) for the highest NO_2_ quartile Adjusted HR: 1.84 (95% CI: 1.57–2.15) for the highest CO quartile	39,819 participants from the Taiwan National Health Insurance Program	International Classification of Diseases 9th
Freeman EE, 2021 [15]	PM_2.5_	Multivariable regression models	Single-pollutant models: Adjusted OR (AMD with visual impairment): 1.52 (95% CI: 1.10–2.09) per IQR increase of PM_2.5_ Multipollutant models: Adjusted OR (AMD with visual impairment): 1.41 (95% CI: 0.96–2.08) per IQR increase of PM_2.5_	30,097 participants from the Canadian Longitudinal Study on Aging	Self-reported data
Patel PJ, 2022 [13]	PM_2.5_, NO_2,_ and PM_10_	Multivariable regression models	Single-pollutant models: Adjusted OR: 1.08 (95% CI: 1.01–1.16) per IQR increase of PM_2.5_ Adjusted OR: 0.99 (95% CI: 0.91–1.08) per IQR increase of NO_2_ Adjusted OR: 0.94 (95% CI: 0.86–1.02) per IQR increase of PM_10_	115,954 participants from the UK Biobank	Self-reported data
Choi YH, 2022 [16]	NO_2_, CO, O_3_, and SO_2_	Survey-logistic regression models	Single-pollutant models: Adjusted OR: 1.24 (95% CI: 1.05–1.46) per IQR increase of NO_2_ Adjusted OR: 1.22 (95% CI: 1.08–1.37) per IQR increase of CO Adjusted OR: 0.83 (95% CI: 0.72–0.95) per IQR increase of O_3_ Adjusted OR: 1.00 (95% CI: 0.93–1.08) per IQR increase of SO_2_	15,115 participants from the Korean National Health and Nutrition Examination	Evaluated by ophthalmologists based on the Wisconsin Age-Related Maculopathy Grading System
Hwang BF, 2022 [8]	PM_2.5_	Time-dependent Cox proportional hazard models	Single-pollutant models: Adjusted HR: 1.19 (95% CI: 1.13–1.25) per IQR increase of PM_2.5_	4,284,128 Taiwanese participants	International Classification of Diseases 9th
Manookin MB, 2022 [18]	O_3_	Multivariable logistic regression models	Single-pollutant models: Adjusted OR: 1.011 (95% CI: 1.003–1.019) per IQR increase of O_3_	9,884,527 participants from the American Intelligent Research in Sight Registry	International Classification of Diseases 9th or 10th
Yan H, 2023 [14]	PM_2.5_	Multivariable logistic regression models	Single-pollutant models: Adjusted OR: 2.602 (95% CI: 1.516–4.468), for the highest PM_2.5_ quartile	36,081 participants from the Chinese Rural Epidemiology for Glaucoma study	International Classification of Diseases 9th
Sun XD, 2024 [17]	O_3_	Multivariable logistic regression models	Single-pollutant models: Adjusted OR: 1.02 (95% CI: 1.01–1.03), for O_3_ concentrations below 110 μg/m^3^ Adjusted OR: 1.68 (95% CI: 1.64–1.71), for O_3_ concentrations above 110 μg/m^3^ Multipollutant models: Adjusted OR: 1.15 (95% CI: 1.13–1.16), for O_3_ concentrations below 110 μg/m^3^ Adjusted OR: 1.66 (95% CI: 1.63–1.69), for O_3_ concentrations above 110 μg/m^3^	624,167 participants from a Chinese hospital-based survey	Evaluated by ophthalmologists based on the Wisconsin Age-Related Maculopathy Grading System

*Abbreviations*: *CI* Confidence interval, *CO* Carbon monoxide, *HR* Hazard ratio, *IQR* Interquartile range, *NO*_*2*_ Nitrogen dioxide, *O*_*3*_ Ozone, *OR* Odds ratio, *PM*_*2.5*_ Particulate matter less than 2.5 μm in diameter, *PM*_*10*_ Particulate matter less than 10 μm in diameter

### Quantitative synthesis

We used the data of single-pollutant models for effect estimates. Two articles calculated and divided air pollution concentrations into four quartiles; we used the data of the highest quartile in the meta-analysis [7, 14]. According to quantitative methods in the review of epidemiologic articles, we ignored the distinctions among the HRs and ORs and calculated the pooled OR [19].

**Fig. 2:**
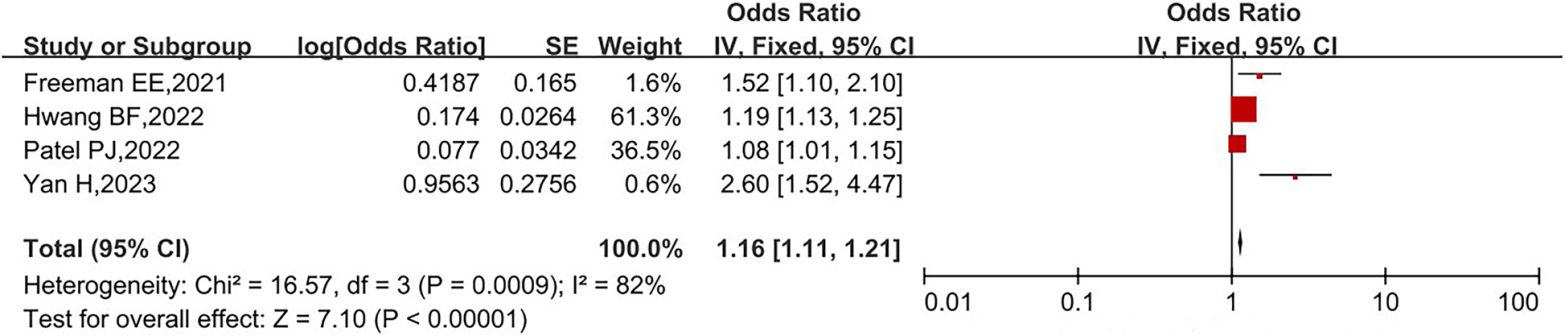
Forest plot of the association between PM_2.5_ and AMD risk. The pooled OR is 1.16 (95% CI: 1.11–1.21, I^2^ = 82%, *P* = 0.0009), demonstrating a positive relationship between PM_2.5_ and AMD. Abbreviations: PM_2.5_, particulate matter less than 2.5 μm in diameter; AMD, age-related macular degeneration; OR, odds ratio; CI, confidence interval; I^2^, inconsistency score

**Fig. 3:**
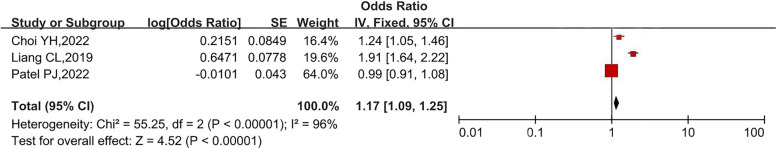
Forest plot of the association between NO_2_ and AMD risk. The pooled OR is 1.17 (95% CI: 1.09–1.25, I^2^ = 96%, *P* < 0.00001), indicating a positive relationship between NO_2_ and AMD. Abbreviations: NO_2_, nitrogen dioxide; AMD, age-related macular degeneration; OR, odds ratio; CI, confidence interval; I^2^, inconsistency score

**Fig. 4:**
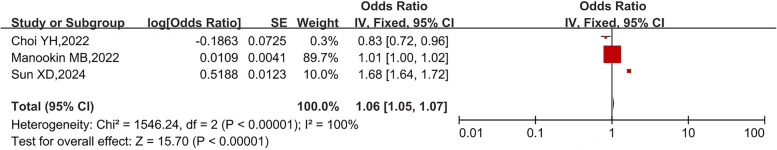
Forest plot of the association between O_3_ and AMD risk. The pooled OR is 1.06 (95% CI: 1.05–1.07, I^2^ = 100%, *P* < 0.00001), indicating a positive relationship between O_3_ and AMD. Abbreviations: O_3_, ozone; AMD, age-related macular degeneration; OR, odds ratio; CI, confidence interval; I^2^, inconsistency score

Figure 2 illustrates the association between PM_2.5_ and AMD; the pooled OR was 1.16 (95% CI: 1.11–1.21, I^2^ = 82%, *P* = 0.0009). Figure 3 shows a forest plot of the outcomes of three studies on the relationship between NO_2_ and AMD; the pooled OR was 1.17 (95% CI: 1.09–1.25, I^2^ = 96%, *P* < 0.00001). Figure 4 illustrates the relationship between O_3_ and AMD; the pooled OR was 1.06 (95% CI: 1.05–1.07, I^2^ = 100%, *P* < 0.00001). All results demonstrated a positive relationship between ambient air pollution and AMD, as well as indicated high heterogeneity.

### Evidence evaluation

Due to the small number of studies eligible for analysis, a statistical evaluation of publication bias was not feasible [20]. NOS was used to assess the quality of the included studies (Additional file 1); one study was classified as having moderate quality [15], whereas the others were classified as having high quality [7, 8, 13, 14, 16–18].

## Discussion

AMD is a progressive retinal disease associated with photoreceptor atrophy and degeneration of the retinal pigment epithelium with a high prevalence and limited therapeutic benefits [4, 5, 21, 22]. The mechanisms underlying AMD are multifactorial. Smoking is a detrimental factor for AMD [23, 24]. Thus, air pollutants, to which the outer eye segment is directly exposed, might also be potential risk factors for eye diseases. Previous studies have shown that air pollution has detrimental effects on ocular surface and increases the risk of dry eye disease and allergic conjunctivitis [25–27]. Recently, researchers have suggested that air pollution may also affect the inner eye segment. The relationships between air pollution and AMD, glaucoma, cataract, and diabetic retinopathy have also been investigated [28–33]. However, the results remain inconclusive. Therefore, we reviewed the existing studies and performed a meta-analysis to clarify this relationship. Notably, various studies have relatively consistently demonstrated a correlation between AMD and PM_2.5_. However, the results of individual studies exploring the exact association between AMD and NO_2_ or O_3_ are contradictory. Our meta-analysis indicated that three air pollutants increased the risk of AMD. Merely one study explored the relationship between SO_2_ and AMD. Additionally, two studies on the association between CO and AMD showed consistent findings that CO increased the AMD risk. For the reasons above, we did not analyse the association between the AMD risk and SO_2_ or CO.

Several potential mechanisms could explain these associations. As chemical components of air pollution, CO, NO_2_, O_3_, SO_2_, and PM_2.5_ share a common biological pathway known to induce oxidative stress and inflammation, which are recognised as AMD risk factors [34, 35]. Moreover, animal studies have demonstrated that PM_2.5_ can impair microvascular function [36]. In the eye, choroidal microcirculation deterioration plays a critical role in AMD [37]. Chua et al. also proposed that exposure to PM_2.5_ is associated with adverse retinal structural features, which may lead to AMD [13, 38]. In addition, PM_2.5_ can cause neurodegenerative diseases, including reduced cognitive function [39, 40], accelerated cognitive decline [41], Parkinson’s disease, and Alzheimer’s dementia [42]. Given that AMD is a neurodegenerative disease, these studies further justify the plausibility of a correlation between AMD and air pollution.

It should be noted that meterological variables have a significant effect on changes in air pollution. Yan et al. reported that combined exposure to PM_2.5_ and atmospheric pressure remarkably increased the risk of AMD, while temperature and humidity acted a weakly antagonistic effect on AMD [14]. Higher temperature is known to cause lower relative humidity. Moreover, both air temperature and atmospheric pressure affect the distribution and concentration of PM_2.5_ [14, 43]. At present, limited studies elucidate the joint effects of meterological factors and ambient air pollution on AMD. Therefore, further studies are needed to clarify this correlation.

Our meta-analysis has certain limitations. First, only a few studies were eligible for inclusion owing to the novelty of this topic. However, most of the included studies had large sample sizes and all were considered credible. Second, the standard definition of AMD was inconsistent among the studies. In addition, the measurement variability of the exposure assessment and the quantitative differences of the exposure extent could bias our findings. Moreover, we used the data of the highest quartile of two articles in the meta analysis and did not standardize the effect size across the studies. The small amount of studies for every air contaminant made subgroup analysis by the exposure level difficult. Finally, other pollutants can affect single-pollutant models. Multipollutant models are less likely to be affected by confounding factors but may be susceptible to other biases.

## Conclusions

The current evidence suggests that ambient air pollutants, such as PM_2.5_, NO_2_, and O_3_, detrimentally affect AMD. Extensive studies are urgently required to investigate additional air pollution and their influence on AMD or other ocular diseases. Further strategies for reducing ambient air pollution exposure are essential for public health, which may ultimately mitigate AMD.

### Supplementary Information

**Additional file 1.** Newcastle-Ottawa Scale for assessing the quality of studies in the meta-analysis. 

## Data Availability

The datasets used and/or analysed during the current study are available from the corresponding author on reasonable request.

## Abbreviations

AMD: Age-related macular degeneration
CI: Confidence interval
CO: Carbon monoxide
HR: Hazard ratio
I^2^: Inconsistency score
NO_2_: Nitrogen dioxide
NOS: Newcastle–Ottawa Scale
OR: Odds ratio
O_3_: Ozone
PM_2.5_: Particulate matter less than 2.5 μm in diameter
PM_10_: Particulate matter less than 10 μm in diameter
SO_2_: Sulphur dioxide
