# Supplementary material for: Association between ambient air pollution and age-related macular degeneration: a meta-analysis
Source: BMC Ophthalmol. 2024 Apr 30;24:202. doi: 10.1186/s12886-024-03465-y (PMC11059589; doi:10.1186/s12886-024-03465-y)
Supplement: Supplementary file 1 — Additional file 1. Newcastle-Ottawa Scale for assessing the quality of studies in the meta-analysis. [file 12886_2024_3465_MOESM1_ESM.pdf]

Study	Selection				Comparability	Exposure			Scores
	Adequate definition of cases	Representativeness of cases	Selection of controls	Definition of controls		Ascertainment of exposure	Same method of ascertainment for cases and controls	Non-response rate	
Liang CL, 2019	★	★	★	★	★★	★	★		8
Freeman EE, 2021		★		★	★★	★	★		6
Patel PJ, 2022		★	★	★	★★	★	★		7
Choi YH, 2022	★	★	★	★	★★	★	★		8
Hwang BF, 2022	★	★	★	★	★★	★	★		8
Manookin MB, 2022	★	★	★	★	★★	★	★		8
Yan H, 2023	★	★	★	★	★★	★	★		8
Sun XD, 2024	★	★	★	★	★★	★	★		8

Additional File 1. Newcastle-Ottawa Scale for assessing the quality of studies in the meta-analysis
